# Identifying Likely Transmission Pathways within a 10-Year Community Outbreak of Tuberculosis by High-Depth Whole Genome Sequencing

**DOI:** 10.1371/journal.pone.0150550

**Published:** 2016-03-03

**Authors:** Alexander C. Outhred, Nadine Holmes, Rosemarie Sadsad, Elena Martinez, Peter Jelfs, Grant A. Hill-Cawthorne, Gwendolyn L. Gilbert, Ben J. Marais, Vitali Sintchenko

**Affiliations:** 1 NSW Mycobacterium Reference Laboratory, Centre for Infectious Diseases and Microbiology Laboratory Services, Institute of Clinical Pathology and Medical Research–Pathology West, Sydney, Australia; 2 Centre for Infectious Diseases and Microbiology–Public Health, Westmead Hospital, Sydney, Australia; 3 Centre for Research Excellence in Tuberculosis and the Marie Bashir Institute for Infectious Diseases and Biosecurity, The University of Sydney, Sydney, Australia; 4 Children’s Hospital at Westmead, Sydney, Australia; 5 School of Public Health, University of Sydney, Sydney, Australia; National Institute of Infectious Diseases, JAPAN

## Abstract

**Background:**

Improved tuberculosis control and the need to contain the spread of drug-resistant strains provide a strong rationale for exploring tuberculosis transmission dynamics at the population level. Whole-genome sequencing provides optimal strain resolution, facilitating detailed mapping of potential transmission pathways.

**Methods:**

We sequenced 22 isolates from a *Mycobacterium tuberculosis* cluster in New South Wales, Australia, identified during routine 24-locus mycobacterial interspersed repetitive unit typing. Following high-depth paired-end sequencing using the Illumina HiSeq 2000 platform, two independent pipelines were employed for analysis, both employing read mapping onto reference genomes as well as *de novo* assembly, to control biases in variant detection. In addition to single-nucleotide polymorphisms, the analyses also sought to identify insertions, deletions and structural variants.

**Results:**

Isolates were highly similar, with a distance of 13 variants between the most distant members of the cluster. The most sensitive analysis classified the 22 isolates into 18 groups. Four of the isolates did not appear to share a recent common ancestor with the largest clade; another four isolates had an uncertain ancestral relationship with the largest clade.

**Conclusion:**

Whole genome sequencing, with analysis of single-nucleotide polymorphisms, insertions, deletions, structural variants and subpopulations, enabled the highest possible level of discrimination between cluster members, clarifying likely transmission pathways and exposing the complexity of strain origin. The analysis provides a basis for targeted public health intervention and enhanced classification of future isolates linked to the cluster.

## Introduction

Nine million new tuberculosis cases were identified in 2013 with an estimated 1.5 million tuberculosis-associated deaths worldwide [1]. The vast majority of cases occur in tuberculosis-endemic areas, where the epidemic is sustained by ongoing transmission, including transmission of drug resistant *Mycobacterium tuberculosis* strains [2]. There is growing appreciation of the need to gain a more thorough understanding of tuberculosis transmission dynamics. While traditional strain typing methods can identify clusters of cases and highlight the role of casual contacts in the transmission of tuberculosis, they lack sufficient resolution to investigate potential paths of transmission [3].

Whole-genome sequencing (WGS) is increasingly used in investigations of community outbreaks of tuberculosis in low and high incidence settings [4][5][6][7][8][9]. WGS offers the prospect of identifying likely sources of infection, assessing drug resistance acquisition and monitoring “real time” strain micro-evolution. The use of single nucleotide polymorphisms (SNPs) to construct phylogenetic trees has allowed for the detailed reconstruction of likely tuberculosis transmission pathways [4][5][6][7][10]. Recent reports highlight possible heterogeneity in *M*. *tuberculosis* populations due to intrapatient microevolution and clonal variants [11][12]. However, the impact of this heterogeneity on the inference of transmission pathways remains controversial and subpopulation analysis requires ‘deep’ sequencing of *M*. *tuberculosis* genomes with coverage exceeding 50-fold.

Australia has one of the lowest tuberculosis incidence rates in the world (6 per 100,000 population per year); immigrant and indigenous populations carry a disproportionate burden of disease [13][14]. More than 85% of cases occur in immigrant populations with the top five countries of origin being India, Vietnam, the Philippines, China and Nepal; areas with significant rates of drug-resistant tuberculosis. Given the low likelihood of multiple exposure and reinfection events, Australia provides an ideal environment to examine transmission pathways within the community, whenever this occurs.

In this study we applied high-coverage WGS to a large cluster of *M*. *tuberculosis* isolates obtained from patients associated with a local tuberculosis cluster in New South Wales (NSW), Australia, in order to clarify likely transmission pathways. We also explored the origin and evolution of the outbreak. The cluster included 23 patients with culture-confirmed pulmonary tuberculosis diagnosed over a period of ten years in NSW [15], despite public health interventions that included active case finding as well as efforts to raise community awareness, trace close contacts and provision of treatment for latent *M*. *tuberculosis* infection. This study enabled division of the cluster into two or three subclades and revealed potential transmission pathways, but still lacked sufficient resolution for certainty regarding individual transmission events.

## Materials and Methods

### *M*. *tuberculosis* isolates

All *M*. *tuberculosis* isolates recovered from culture-confirmed cases of tuberculosis in NSW are forwarded to the Mycobacterium Reference Laboratory at the Institute of Clinical Pathology and Medical Research (ICPMR). In addition to drug susceptibility testing, prospective 24-locus mycobacterial interspersed repetitive unit (MIRU-24) typing is performed on all specimens [16]. MIRU-24 typing usually identifies cases as singletons or small clusters limited to one generation of transmission [17]. Isolates of *M*. *tuberculosis* are stored in glycerol at -80°C. Of 23 strains that formed a single MIRU cluster identified over a 10-year period (2003–2012), 22 were successfully subcultured from storage on Middlebrook 7H10 medium and sequenced. All isolates were recovered from adult patients with pulmonary tuberculosis and were phenotypically susceptible to first-line anti-tuberculosis drugs (rifampicin, isoniazid, pyrazinamide and ethambutol). Ethics approval was not required, as sequencing and analysis were performed as part of routine public health operations for outbreak investigation.

### DNA extraction, library preparation and sequencing

*M*. *tuberculosis* DNA was harvested from colonies growing on Middlebrook 7H10 medium using a chloroform-isoamyl alcohol method [18]. WGS was performed by the Australian Genomics Research Facility (www.agrf.org.au) using the Illumina HiSeq 2000 platform, producing 100 bp paired-end reads. Depth of coverage across the 22 libraries ranged from 120- to 690-fold (median 357-fold), and insert size from 185 to 260 bp (median 222 bp).

### Bioinformatic analysis

Two independent pipelines were employed to analyse WGS reads. The first pipeline utilized Qiagen *CLC Genomics Workbench* 7.0 (CLC Bio, Aarhus, Denmark) for *de novo* assembly of the earliest isolate, c2(2003). The resulting contigs were concatenated into H37Rv genomic order using *Mauve* v2.3.1 [19]. Read mapping was performed against H37Rv (NC_000962.2, Genbank accession AL123456.2 [20]), and against the concatenated *de novo* assembly of c2(2003). Variants were called using *CLC Genomics Workbench* and *LoFreq* [21], for enhanced detection of mixed populations that might have been present *in vivo*. Variant filtering was set to only include variants that were present at genomic positions that had quality scores of ≥20, at least ≥10-fold read depth coverage including at least one read on both forward and reverse strands, and were present at frequencies of either ≥75% or ≥90% of mapped reads. A subsequent filter, applied to *CLC* and *LoFreq* variants, removed all variants with non-unique mapping or low coverage in one or more of the isolates [see supporting information]. Variants shared between all isolates were excluded from further analysis. Variants that discriminated within the cluster were individually confirmed by visual inspection of read alignment. For the construction of maximum parsimony trees, single-nucleotide insertions and deletions were arbitrarily treated as equivalent to a single-nucleotide polymorphism, and sites with no variation were discarded from further analysis.

The second analysis was performed using reads that underwent error-correction with *BayesHammer* [22]. Mapping of error-corrected reads was performed using *bwa-mem* [23]. Spoligotype was inferred from corrected reads with *SpolPred* [24][25]. Lineage was assigned by mapping reads against previously described regions of difference [26][27]. Variants were called on all samples in parallel using *freebayes* [28]. Structural variants were examined with *DELLY* [29]. *De novo* assemblies were generated using *SPAdes* [30]. A pseudo-genome with reduced repetitive elements, H37Rv-RRE, was created as a reference for multiple sequence alignment from the *M*. *tuberculosis* H37Rv genome, by substituting gaps for all elements annotated as PE/PPE/PGRS and *cysA* genes, insertion sequences, transposases and prophage components (6.3% of the H37Rv genome replaced with gaps). Contigs of 1000 bp or more were matched and sorted into H37Rv order using r2cat [31], aligned using mauve [19] and then concatenated (with gaps inserted at junctions) into a pseudo-genome. Reads were remapped against the corresponding pseudo-genome using *bwa-mem* [23], and all variant sites were masked as an additional error correction step. Multiple sequence alignment of corrected cluster pseudo-genomes, the H37Rv-RRE pseudo-genome and 8 other reference genomes from lineage 4 was performed using *progressiveMauve* [32]. All columns in the resulting multiple sequence alignment that contained a gap were deleted, as available phylogenetic models are unable to take into consideration insertions or deletions.

### Relationships between genomes and probable transmission pathways

Concatenated variant profiles were used to infer patterns of microevolution using the maximum parsimony method implemented in *MEGA-6* [33]. Bayesian inference trees were produced using *BEAST* (v1.8.0) [34], with diagnostic specimen collection dates used to constrain node ages, and the HKY substitution model over 100,000,000 generations, with assessment for steady state after completion. Strict constant, strict skyline, relaxed constant and relaxed skyline clock and population models were used, and stepping stone marginal likelihood was estimated after completion and compared between models (see supporting information for additional parameters).

Variant data was also used to construct graphs of potential transmission which incorporated some or all of the following assumptions: (i) variant files contain no homoplasy resulting from convergence (the appearance of the same variant in two or more separate isolates) or reversion (return to the ancestral state by backwards mutation); (ii) transmission can only occur forwards in time (patients detected later cannot be a source for infections that were diagnosed earlier); (iii) no further introductions of *M*. *tuberculosis* into the cluster after 2003 (i.e. the absence, since the introduction of routine typing, of multiple introductions of *M*. *tuberculosis* from individuals who were not identified as part of the cluster); (iv) all subsequent cases arose within 3 years of a transmission event.

## Results

### Genotyping and lineage assignment

**Fig 1:**
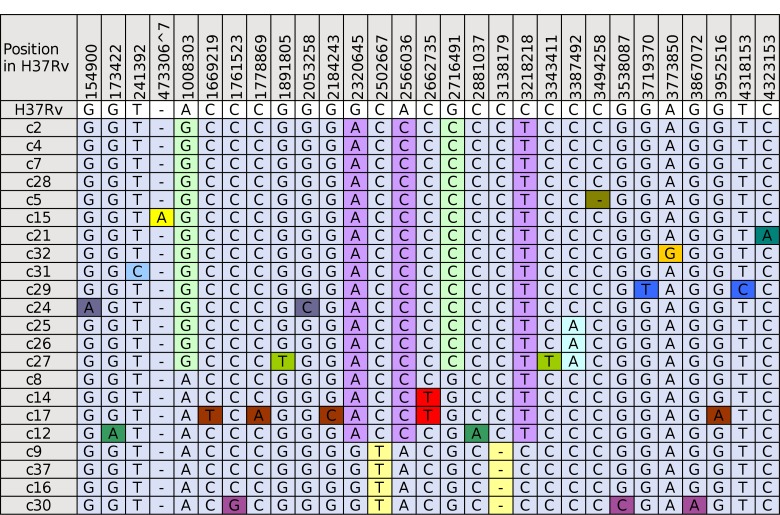
Matrix of M. tuberculosis variants associated with the outbreak. Locations of all SNPs and indels found in the 22 isolates are shown in the colour-coded matrix. Deletions, or the absence of an insertion, are indicated with a single dash (-). Genome locations of variants are given for the reference strain H37Rv. Colour-coding of variants is based on differences from H37Rv, with all variants appearing in that isolate coded the same colour. This colour is then maintained when these variants appear in subsequent isolates, to help visualise patterns of SNP accumulation.

In total 23 patient isolates were identified with identical MIRU-24 loci and spoligotype (MIRU-12: 23’3425153322; MIRU-24: 242324223342; spoligotype: 777737777740371). The isolate from the earliest clinical case identified as part of the cluster (c1) could not be subcultured. All 22 subcultured isolates (96% of the eligible outbreak cases, listed in Fig 1) were spread between 2003 and 2012. Short reads from whole-genome sequencing of these 22 isolates were deposited as European Nucleotide Archive accession PRJEB11859. The isolates were confirmed to be members of *M*. *tuberculosis* complex lineage 4 by region of difference analysis: they possessed the TbD1 deletion, a 7bp deletion in *pks15*, and no deletion at RD105 and RD750 [26]. No deletions were found at RD115, RD174, RD182, RD193, RD724 or RD761 to facilitate sub-classification within lineage 4. Two isolates came from a single patient who experienced tuberculosis recurrence (c15 and c26), the second episode arising two years after successful completion of directly observed therapy.

### Identification of fixed SNPs and indels

WGS achieved high genome coverage, with sequencing reads covering over 98% of both reference genomes (H37Rv and *de novo* assembled c2(2003) reference genomes). Both bioinformatic pipelines, assessing variants identified in 90% or more of mapped reads, identified the same set of variants in the 22 isolates sequenced. No structural variants that could discriminate between members of the cluster were found. SNPs and single base insertions or deletions (indels) are listed in Fig 1.

The total number of variants detected was low. Variant analyses detected 29 variants, including 26 SNPs, one insertion and two deletions, which show accumulation of variation within the cluster (Fig 1). All variants were present in both independent variant analyses. The most distant isolates were separated by 13 variants (from c30 to either c17 or c27; c17 and c27 are separated by 10 variants). Nine of the isolates contained no detectable variants from at least one other isolate and formed three genetically indistinguishable sub-clusters. The other thirteen contained one or more unique variants that were not present in any of the other isolates. Sequenced isolates could be placed into 14 groups using SNPs alone, or 16 groups using SNPs and indels. These groups were used to model potential transmission pathways.

### Likely transmission pathways from unrooted analysis

**Fig 2:**
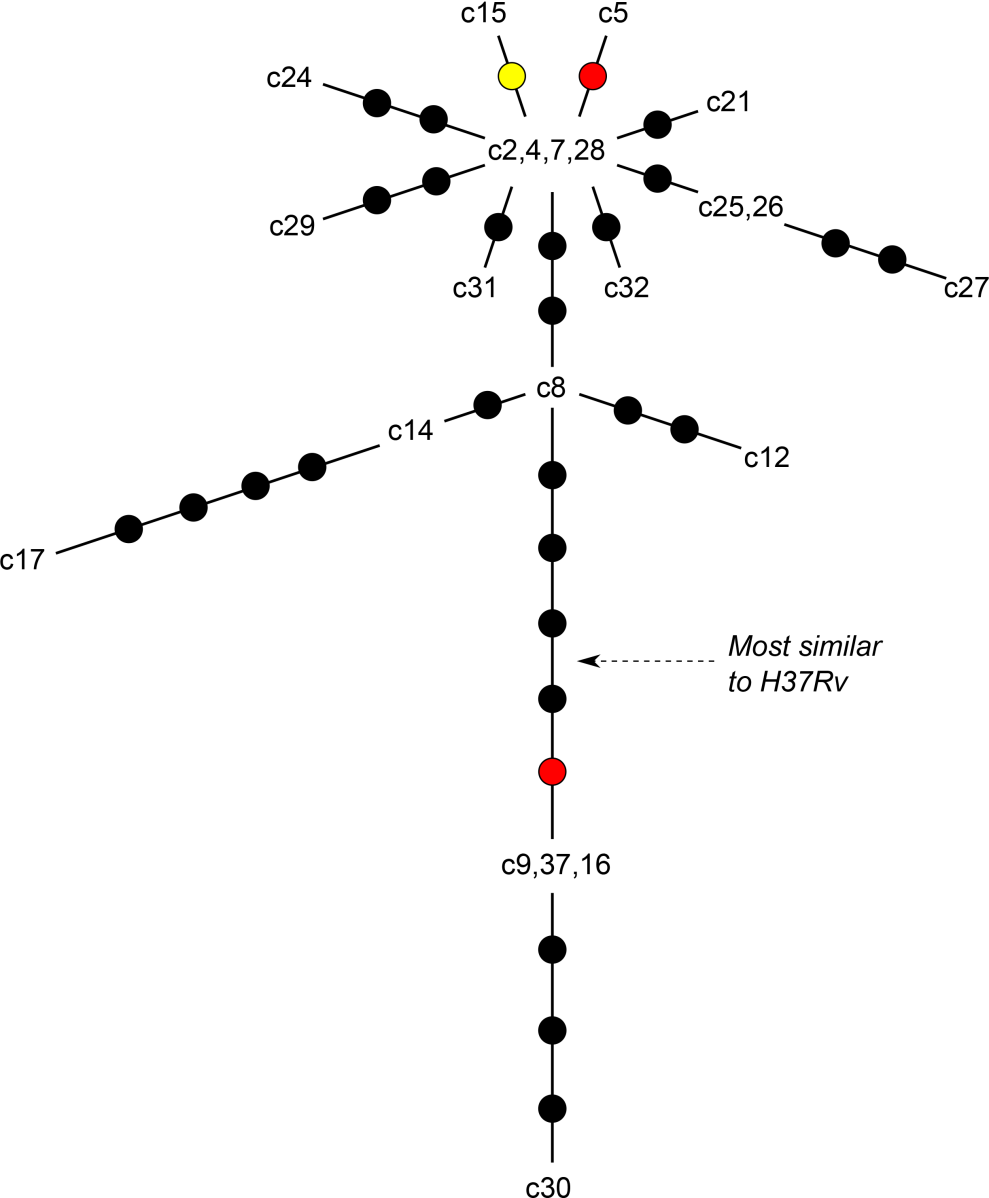
Unrooted maximum parsimony tree of variants. The tree depicts the relative genetic distances between cluster isolates, estimated from maximum parsimony analyses performed on concatenated variants. Isolates that were genetically indistinguishable based on variant analyses are grouped together. Branch lengths are relative to the number of variants separating each isolate; individual SNPs, insertions, and deletions are represented by black, yellow, and red dots, respectively.

**Fig 3:**
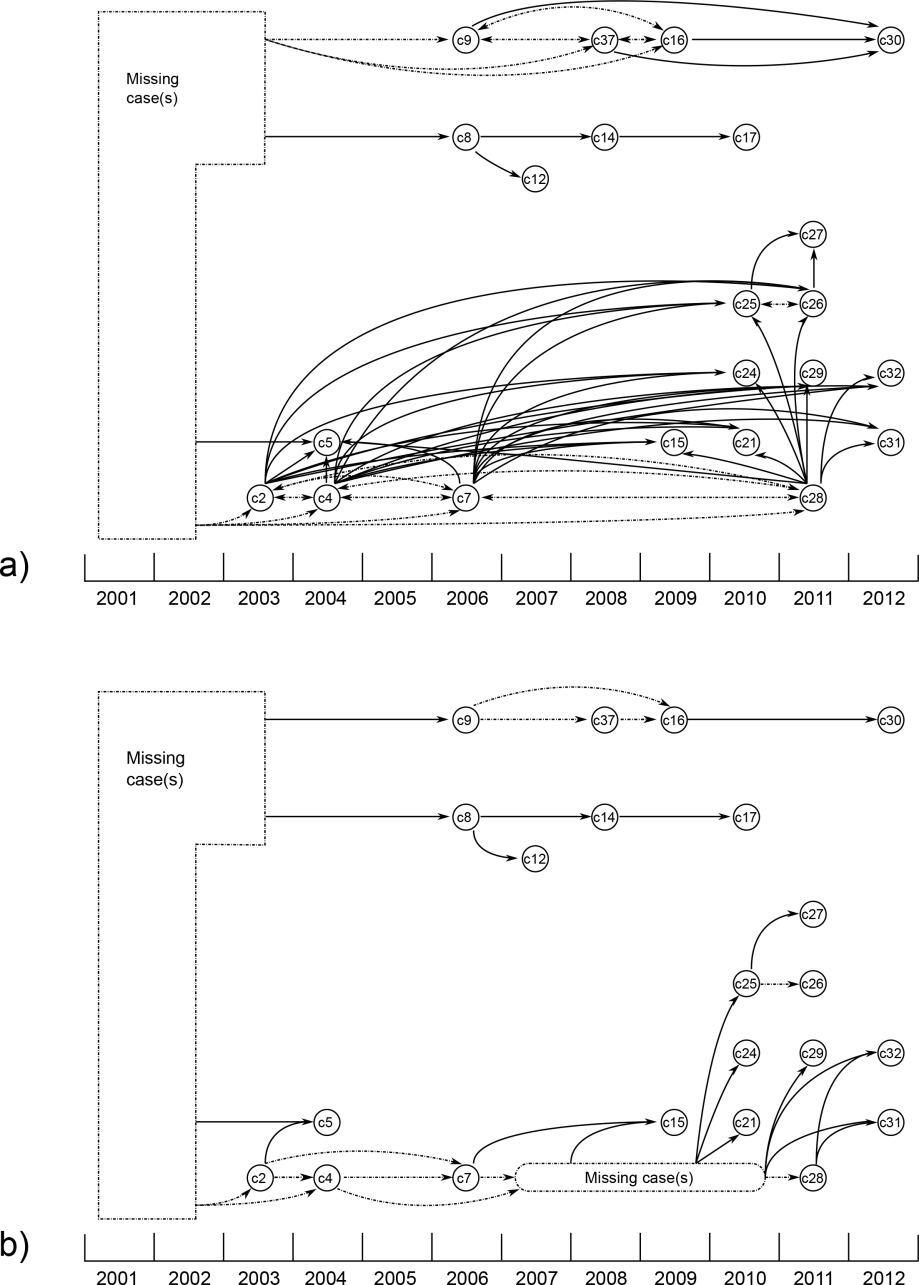
Transmission pathways derived from unrooted maximum parsimony tree. Each circle (or node) represents a sequenced isolate. Nodes are positioned according to the year the original specimen was collected. Dashed lines connect nodes that are indistinguishable based on variant analyses. Solid lines indicate at least one observed variant between two nodes. Putative transmission events are indicated by arrows based on: (a) variant analyses and assumptions of no homoplasy and no introductions after 2003; and (b) variant analyses, no homoplasy, no introductions after 2003 and further epidemiological assumptions. The further epidemiological assumptions applied are (i) chronological transmission; (ii) transmission could not occur between cases that were diagnosed within 6 months of each other; and (iii) secondary cases arose within three years of exposure to a possible source case. The application of these assumptions indicated that at least two unidentified cases would have been required to sustain cluster transmission (“Missing Case(s)” boxes). However if, for example, the insertion found in the c15 library had arisen after transmission, then even with these assumptions no missing cases would be required later than 2003.

Potential pathways of transmission were inferred from a maximum parsimony tree constructed using SNPs, deletions and insertions (Fig 2). The lines represent potential ancestral relationships that may be associated with transmission pathways, but the directionality of transmission events could not be established. Fig 3A shows possible transmission pathways that are compatible with the variants detected using the first read mapping pipelines and a set of baseline assumptions. Fig 3B reflects possible transmission pathways constrained by three additional assumptions: (i) chronological transmission; (ii) that transmission did not occur between cases that were diagnosed within 6 months of each other (unlikely provided all cases were diagnosed soon after symptom onset in a low incidence country with access to specialist care); and (iii) that secondary cases arose within three years of source case exposure. The assumptions made in order to construct Fig 3 are likely to be true in general, but the possibility that they were breached in one or more instances of transmission will increase with cluster size.

### Additional information from low frequency variants

**Fig 4:**
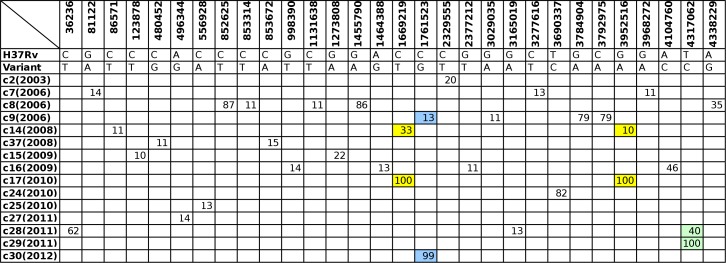
Low Frequency Variant Detection. LoFreq was used to detect SNPs present at frequencies ≥ 10%. The top row indicates the SNP position in the reference genome H37Rv, the second row shows the nucleotide present in H37Rv, and the third row shows the variant nucleotide identified by LoFreq. The matrix indicates the frequencies of the SNPs detected in the isolates shown.

An additional five variants were detected by relaxing the variant-calling threshold from 90% to 75% of mapped reads (S1 Fig). This lower threshold discriminated the 22 isolates into 18 groups. Six isolates in two of these groups had no unique variants (relative to at least one other isolate). Detailed assessment of variants present in less than 75% of reads identified a number of extra variants that were excluded from the initial analysis (S1 Table). Most of these subpopulations were unique to single libraries with uncertain relevance. However the presence of some minority variants (shown in Fig 4) supports a postulated transmission link between two cases in the cluster. Isolate c14(2008) possessed two subpopulation variants, present in 10% and 33% of reads, that were both unanimous in the subsequent isolate c17(2010); isolate c28 (2011) possessed a variant present in 40% of reads that was subsequently unanimous in isolate c29(2011); and isolate c9(2006) displayed a variant in 13% of reads that was unanimous in isolate c30(2012). In all three cases a variant found in a minority of reads in an earlier isolate became fixed in a closely related subsequent isolate, which would be consistent with transmission of that subpopulation.

### Reference-free detection of variants

Contigs constructed by *de novo* assembly were 97–98% homologous with the complete H37Rv reference genome. Gap-free multiple sequence alignment of the 22 *de novo*-assembled genomes, the H37Rv-RRE pseudogenome and 8 other reference genomes representing *M*. *tuberculosis* complex lineage 4, contained 3,798,786 columns (92% of H37Rv-RRE, and 86% of the complete H37Rv genome). Within this alignment, 24 columns showed variation between members of the cluster, which is fewer than the 34 variants found using standard read mapping pipelines. The 10 variants not detected using this approach were indels, or occurred at sites that did not have unanimous base calls across all members of the cluster and were thus excluded from the analysis.

### Bayesian inference phylogeny of the cluster within Lineage 4

**Fig 5:**
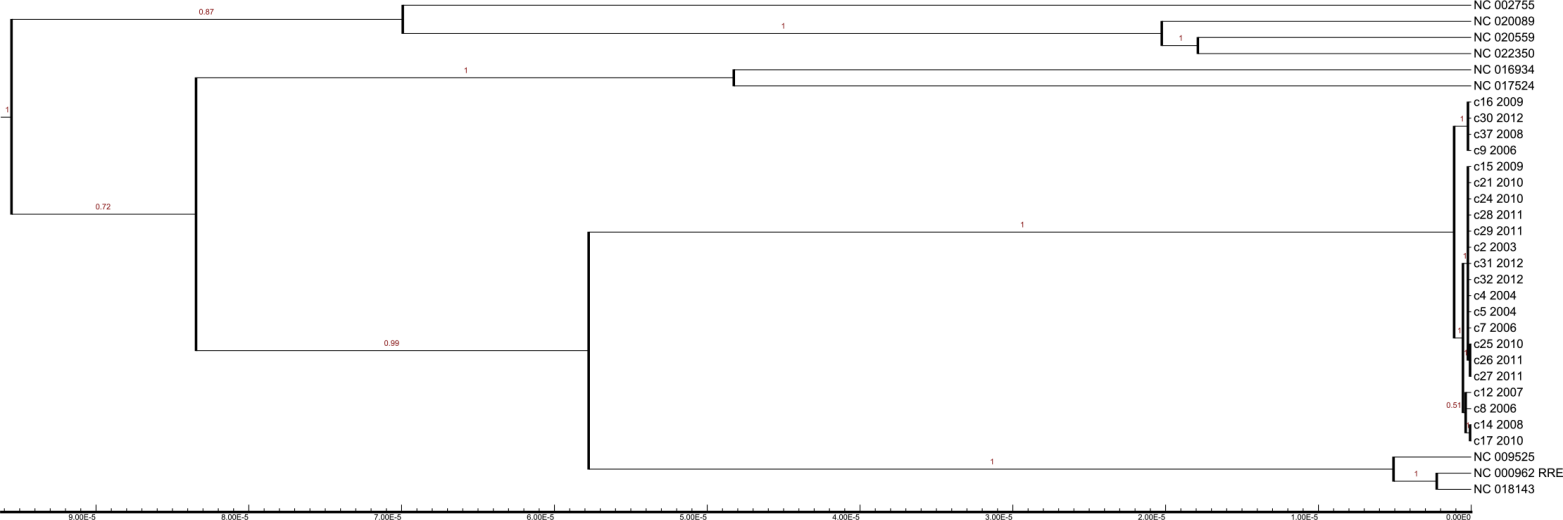
Bayesian inference tree from multiple sequence alignment of *de novo*-assembled cluster genomes and reference genomes. A multiple sequence alignment of the H37Rv genome with repetitive elements censored (NC_000962.RRE), eight other lineage 4 reference genomes and the de novo assembled cluster libraries was used to generate Bayesian inference trees using BEAST. A consensus tree using relaxed clocks and the coalescent skyline population model is shown, with branch labels showing the probability of those subclades appearing in the sampled trees; substitutions per site appear on the y-axis. Subclades that appeared in less than half of the sampled trees are not shown. The SNPs that determine the characteristics of this tree are a subset of the variants shown in Fig 1.

Fig 5 shows a constant-population model Bayesian inference tree derived from whole genome multiple sequence alignment, using a fixed molecular clock, and visualizes the cluster in the context of nine lineage 4 reference genomes. BEAST MCMC stepping stone marginal likelihood estimation found modestly superior likelihood from the relaxed skyline model, but differences in tree topology were minimal. The tree discriminated 5 subclades within the cluster with high posterior probability (p>0.95), and a further subclade with moderate probability (p>0.5). Apart from the main 11-member subclade including c2, four isolates (c9, c16, c30 and c37) were categorized as a distinct clade, most likely representing a separate chain of transmission. Another four isolates (c8, c12, c14 and c17) show indeterminate connection to the main clade (probability 0.51); it is possible that the common ancestor of these four isolates precedes the chain of transmission in the main clade. Three isolates (c25, c26 and c27) form a subclade that appears to have arisen from the main clade. The structure of the phylogeny suggests unknown or missing cases.

## Discussion

Our findings demonstrate the value and complexity of deep WGS for the reconstruction of likely transmission pathways during community outbreaks of tuberculosis [4][7][8][9]. It illustrates the potential utility of including indels and genomic subpopulations when assessing likely chains of transmission. However, analyses were dependent on the bioinformatic pipelines used and the assumptions underpinning evolutionary models. Tuberculosis presents particular challenges, since variable periods of clinical latency result in non-linear transmission chains. In addition, *M*. *tuberculosis* has a propensity towards homoplasy in certain genetic markers [35][36], may display inconsistent molecular clocks in different lineages [37] and has a low mutation rate per unit of time, necessitating long intervals to observe variation [38][39].

Therefore our approach used two or more unrelated and peer-reviewed algorithms to enable some degree of internal verification. The generation of a maximum likelihood or Bayesian inference tree (including appropriate reference genomes) provides additional context that allows more reliable interpretation of variant data. Although emphasis should be placed on identifying the most likely transmission pathways, alternative scenarios also require consideration. Triangulation with epidemiological data and detailed mapping of social contacts increases confidence that the genomic data have been interpreted correctly.

The uncertainties identified make it impossible to characterize transmission pathways with complete confidence. Fig 3B provides the simplest overview of likely transmission pathways. It is important to note that one small group of isolates (comprising c9, c16, c30 and c37) appears to have a shorter genetic distance (by 5 SNPs) than the large group (comprising c2, c4, c5, c7, c15, c21, c24, c25, c26, c27, c28, c29, c31, c32) from their common ancestor with H37Rv. Thus, in the absence of homoplasy the small group is unlikely to have arisen from the larger group. Another four isolates (c8, c12, c14 and c17) have an intermediate genetic distance from the common ancestor, as illustrated by the Bayesian inference tree (Fig 5). The continued emergence of new cases in the cluster, despite concerted efforts to detect and treat latently infected individuals, implies the presence of undiagnosed infectious cases, exemplified in Fig 3B.

Two of the indels introduced important layers of discrimination, by differentiating two isolates from the monophyletic group containing c2(2003) and signifying that these two isolates are less likely to have been sources of onward transmission. Furthermore, the presence of a unique insertion in the c15(2009) library relative to c26(2011), which was obtained from the same patient more than one year after treatment was completed and deemed successful (Sue Devlin, personal communication, July 2014), is consistent with c26(2011) originating after reinfection from contact c25(2010). An alternate explanation is relapse with an “archived” strain that lacked the insertion found in c15(2009) and possessed the subclade SNP shared with c25(2010) and c27(2011), perhaps implying origin of this subclade during the relapse [7][10]. Note that the insertion detected in the c15 library occurred in a repetitive region (Rv0393, S2 Table), increasing the chance that the insertion arose during library preparation.

The heterogeneity of *M*. *tuberculosis* genomes observed suggests that several of the clinical specimens contained distinct subpopulations. Therefore, the selection of SNP profiles based on a single frequency threshold could bias transmission pathway prediction. For example, if transmission chains were inferred based on SNP profiles generated using the variant frequency threshold of ≥90% the isolates c8 and c9 both formed potential nodes of ongoing transmission (S2 Fig); however, they would be excluded from these central positions in their respective transmission chains using a ≥ 75% cut-off. The two additional SNPs identified in c8 were both found in ~87% of reads, suggesting a minority subpopulation without these two variants. The same could be assumed for c9, where the additional SNPs were found at frequencies of ~80%. In both cases, the genomic profiles of the subpopulations without these lower-frequency variants form nodes within growing transmission chains, while the subpopulations containing the additional variants are likely to represent subpopulations that were present in the host but not transmitted, or subpopulations that arose *in vitro*. SNP frequencies detected here reflect the relative abundances of profiles in the material used for sequencing, collected after *in vitro* subculture, and do not necessarily reflect their abundance within the host.

Use of a reference sequence reduces the potential for sequencing errors and false variant calls, compared with *de novo* assembly; however, mapping against a reference genome introduces a bias towards the reference sequence that can result in false homology [40]. To minimize this bias reads were mapped against H37Rv, but also against a *de novo* assembly based on the earliest available member of the cluster, theoretically providing the optimal “point of departure”. Although this approach should limit any bias introduced by sub-optimal reference selection, it could instead lead to overestimation of the similarity between members of the cluster. Methods such as simultaneous *de novo* assembly of a population [41] and use of a reference-independent method to call variants [42] have been designed to avoid these issues, but these approaches have not yet fully matured. The reference-free approach described in the present paper uses established tools and algorithms, but requires multiple correction steps to maintain specificity (to prevent false variants arising due to *de novo* assembly errors, or misalignment during the preparation of contigs or multiple sequence alignment) at the cost of reduced sensitivity (in the best case, inability to detect indels).

Another important insight gained is the recognition that some cases in a MIRU-24 cluster may not share an immediate common ancestor. The group containing c2, although larger and identified earlier, is unlikely to be the ancestral source of the group containing c9, c16, c30 and c37. This is not unexpected and suggests that there may be a common ancestor that preceded routine molecular typing or was not diagnosed. An additional complicating factor to consider is the fact that although *M*. *tuberculosis* organisms found in a single granuloma are typically descended from a single infecting microbe [43], mixed infection can arise from multiple infection events, as implied by the occurrence of simultaneous infection with two unrelated strains [44]. Hence it is possible for *M*. *tuberculosis* infection to be caused by a population of closely (and sometimes distantly) related lineages. This mixture may survive the process of expectoration, culture, storage, subculture and library preparation involved in diagnosis and subsequent WGS. However, the ratios will not be preserved without additional measures that were not taken in this study, and so the mixtures detected may not be representative of processes *in vivo*. In our study, the variants found using a more relaxed 75% cut-off and using LoFreq did not contradict those found using more stringent requirements, but two instances of “foreshadowed” mutations suggest that the analysis of subpopulation variants requires further research. To facilitate this, material for WGS should be obtained after as few subcultures as possible (e.g. directly from the primary MGIT bottle).

## Conclusions

Despite the limitations discussed, it is noteworthy that the findings from high-coverage WGS analysis, using two independent bioinformatic pipelines, correlate well with field observations and social network analysis [15]. Two major branches of transmission identified by WGS correspond to distinct geographical regions of New South Wales. Several of the postulated transmission chains within the subclade containing c2(2003) were supported by family and close-contact links. Variant profiles were also able to shed light on some previously unsuspected links within this cluster, such as case c8(2006), whose connections to the cluster had been difficult for the TB control team to establish. A particular strength of the study is the near completeness of identification of cluster members, with good access to clinical care, a reliable laboratory referral system with routine MIRU typing, and regular cross-checking between the clinical and laboratory teams of new tuberculosis cases identified.

In conclusion, deep sequencing of *M*. *tuberculosis* genomes can provide additional resolution for the delineation of likely transmission pathways during community outbreaks. Using a combination of variants, including SNPs, indels and subpopulations, for maximal resolution seems important given the slow molecular clock of *M*. *tuberculosis*. WGS enables better targeted public health interventions through outbreak confirmation, identifying likely transmission chains, as well as revealing unrecognised sources of transmission.

## Supporting Information

S1 Fig Variant matrix of cluster isolates using 75% threshold. Locations of all SNPs and indels found in ≥75% of reads. Deletions, or the absence of an insertion, are indicated with a single dash (-). Genome locations of variants are given for the reference strain H37Rv (second row). The top row indicates the percentage of reads containing the variant. Colour coding of variants is based on the earliest isolate in which they first appeared; with all variants appearing in that isolate coded the same colour. This initial colour is then maintained when these variants appear in subsequent isolates, in order to visualise patterns of SNP accumulation.
(PDF)

S2 Fig Impact of SNP detection thresholds on potential transmission pathways. Differences in the transmission links for isolates c8 and c9 based on SNP profiles generated using (A) 90% SNP frequency threshold, corresponding to Fig 1, and (B) 75% SNP frequency threshold, corresponding to S1 Fig. The relative frequencies of SNPs detected suggested that several of the isolates in this cluster contained distinct subpopulations. Isolates c8 and c9 are shown to contain multiple variant subpopulations (C). One subpopulation forms part of a transmission pathway while the other subpopulation(s) do not.
(PDF)

S1 Table LoFreq SNPs (frequencies between 10 and 75%). (PDF)

S2 Table Annotation of variants using Tuberculist [45]. (PDF)

## References

[1] World Health Organization. Global tuberculosis report 2014 [Internet]. Geneva, Switzerland: World Health Organization; 2014 Available: http://www.who.int/tb/publications/global_report/en/.

[2] Marais BJ, Mlambo CK, Rastogi N, Zozio T, Duse AG, Victor TC, et al Epidemic Spread of Multidrug-Resistant Tuberculosis in Johannesburg, South Africa. J Clin Microbiol. 2013;51: 1818–1825. 10.1128/JCM.00200-13 23554196 PMC3716102

[3] Wilson DJ. Insights from Genomics into Bacterial Pathogen Populations. PLoS Pathog. 2012;8: e1002874 10.1371/journal.ppat.1002874 22969423 PMC3435253

[4] Gardy JL, Johnston JC, Ho Sui SJ, Cook VJ, Shah L, Brodkin E, et al Whole-genome sequencing and social-network analysis of a tuberculosis outbreak. New Engl J Med. 2011;364: 730–739. 10.1056/NEJMoa1003176 21345102

[5] Kato-Maeda M, Ho C, Passarelli B, Banaei N, Grinsdale J, Flores L, et al Use of Whole Genome Sequencing to Determine the Microevolution of Mycobacterium tuberculosis during an Outbreak. PLoS ONE. 2013;8: e58235 10.1371/journal.pone.0058235 23472164 PMC3589338

[6] Török ME, Reuter S, Bryant J, Köser CU, Stinchcombe SV, Nazareth B, et al Rapid Whole-Genome Sequencing for Investigation of a Suspected Tuberculosis Outbreak. J Clin Microbiol. 2013;51: 611–614. 10.1128/JCM.02279-12 23175259 PMC3553910

[7] Walker TM, Ip CL, Harrell RH, Evans JT, Kapatai G, Dedicoat MJ, et al Whole-genome sequencing to delineate Mycobacterium tuberculosis outbreaks: a retrospective observational study. Lancet Infect Dis. 2013;13: 137–146. 10.1016/S1473-3099(12)70277-3 23158499 PMC3556524

[8] Luo T, Yang C, Peng Y, Lu L, Sun G, Wu J, et al Whole-genome sequencing to detect recent transmission of Mycobacterium tuberculosis in settings with a high burden of tuberculosis. Tuberculosis. 2014;94: 434–440. 10.1016/j.tube.2014.04.005 24888866 PMC4409578

[9] Smit PW, Vasankari T, Aaltonen H, Haanperä M, Casali N, Marttila H, et al Enhanced tuberculosis outbreak investigation using whole genome sequencing and IGRA. Eur Respir J. 2015;45: 276–279. 10.1183/09031936.00125914 25323236

[10] Bryant JM, Harris SR, Parkhill J, Dawson R, Diacon AH, van Helden P, et al Whole-genome sequencing to establish relapse or re-infection with Mycobacterium tuberculosis: a retrospective observational study. Lancet Respir Med. 2013;1: 786–792. 10.1016/S2213-2600(13)70231-5 24461758 PMC3861685

[11] Merker M, Kohl TA, Roetzer A, Truebe L, Richter E, Rüsch-Gerdes S, et al Whole Genome Sequencing Reveals Complex Evolution Patterns of Multidrug-Resistant Mycobacterium tuberculosis Beijing Strains in Patients. PLoS ONE. 2013;8: e82551 10.1371/journal.pone.0082551 24324807 PMC3855793

[12] Pérez-Lago L, Comas I, Navarro Y, González-Candelas F, Herranz M, Bouza E, et al Whole Genome Sequencing Analysis of Intrapatient Microevolution in Mycobacterium tuberculosis: Potential Impact on the Inference of Tuberculosis Transmission. J Infect Dis. 2014;209: 98–108. 10.1093/infdis/jit439 23945373

[13] Bareja C, Waring J, Stapledon R, Toms C, Douglas P, National Tuberculosis Advisory Committee. Tuberculosis notifications in Australia, 2011. Commun Dis Intell. 2014;38: 356–368. 10.33321/cdi.2014.38.57 25631599

[14] Lumb R, Bastian IB, Jelfs PJ, Keehner TJ, Pandey SK, Sievers A. Tuberculosis in Australia: bacteriologically-confirmed cases and drug resistance, 2011: A report of the Australian Mycobacterium Reference Laboratory Network. Commun Dis Intell. 2014;38: 369–375. 10.33321/cdi.2014.38.58 25631600

[15] Devlin S, Passmore E. Ongoing transmission of tuberculosis in Aboriginal communities in NSW. New South Wales Public Health Bull. 2013;24: 38–42. 10.1071/NB12113 23849029

[16] Supply P, Allix C, Lesjean S, Cardoso-Oelemann M, Rüsch-Gerdes S, Willery E, et al Proposal for Standardization of Optimized Mycobacterial Interspersed Repetitive Unit-Variable-Number Tandem Repeat Typing of Mycobacterium tuberculosis. J Clin Microbiol. 2006;44: 4498–4510. 10.1128/JCM.01392-06 17005759 PMC1698431

[17] Gurjav U, Jelfs P, McCallum N, Marais BJ, Sintchenko V. Temporal dynamics of Mycobacterium tuberculosis genotypes in New South Wales, Australia. BMC Infect Dis. 2014;14: 455 10.1186/1471-2334-14-455 25149181 PMC4262242

[18] van Soolingen D, Hermans PW, Haas PE de, Soll DR, van Embden JD. Occurrence and stability of insertion sequences in Mycobacterium tuberculosis complex strains: evaluation of an insertion sequence-dependent DNA polymorphism as a tool in the epidemiology of tuberculosis. J Clin Microbiol. 1991;29: 2578–2586. 1685494 10.1128/jcm.29.11.2578-2586.1991 PMC270376

[19] Darling ACE, Mau B, Blattner FR, Perna NT. Mauve: Multiple Alignment of Conserved Genomic Sequence With Rearrangements. Genome Res. 2004;14: 1394–1403. 10.1101/gr.2289704 15231754 PMC442156

[20] Cole ST, Brosch R, Parkhill J, Garnier T, Churcher C, Harris D, et al Deciphering the biology of Mycobacterium tuberculosis from the complete genome sequence. Nature. 1998;393: 537–544. 10.1038/31159 9634230

[21] Wilm A, Aw PPK, Bertrand D, Yeo GHT, Ong SH, Wong CH, et al LoFreq: a sequence-quality aware, ultra-sensitive variant caller for uncovering cell-population heterogeneity from high-throughput sequencing datasets. Nucleic Acids Res. 2012;40: 11189–11201. 10.1093/nar/gks918 23066108 PMC3526318

[22] Nikolenko SI, Korobeynikov AI, Alekseyev MA. BayesHammer: Bayesian clustering for error correction in single-cell sequencing. BMC Genomics. 2013;14: S7 10.1186/1471-2164-14-S1-S7 PMC3549815 23368723

[23] Li H. Aligning sequence reads, clone sequences and assembly contigs with BWA-MEM. ArXiv13033997 Q-Bio. 2013; Available: http://arxiv.org/abs/1303.3997.

[24] Kamerbeek J, Schouls L, Kolk A, van Agterveld M, van Soolingen D, Kuijper S, et al Simultaneous detection and strain differentiation of Mycobacterium tuberculosis for diagnosis and epidemiology. J Clin Microbiol. 1997;35: 907–914. 9157152 10.1128/jcm.35.4.907-914.1997 PMC229700

[25] Coll F, Mallard K, Preston MD, Bentley S, Parkhill J, McNerney R, et al SpolPred: rapid and accurate prediction of Mycobacterium tuberculosis spoligotypes from short genomic sequences. Bioinformatics. 2012;28: 2991–2993. 10.1093/bioinformatics/bts544 23014632 PMC3496340

[26] <Gagneux S, DeRiemer K, Van T, Kato-Maeda M, Jong BC de, Narayanan S, et al Variable host–pathogen compatibility in Mycobacterium tuberculosis. Proc Natl Acad Sci U S A. 2006;103: 2869–2873. 10.1073/pnas.0511240103 16477032 PMC1413851

[27] Brosch R, Gordon SV, Marmiesse M, Brodin P, Buchrieser C, Eiglmeier K, et al A new evolutionary scenario for the Mycobacterium tuberculosis complex. Proc Natl Acad Sci. 2002;99: 3684–3689. 10.1073/pnas.052548299 11891304 PMC122584

[28] Garrison E, Marth G. Haplotype-based variant detection from short-read sequencing. ArXiv12073907 Q-Bio. 2012; Available: http://arxiv.org/abs/1207.3907

[29] Rausch T, Zichner T, Schlattl A, Stütz AM, Benes V, Korbel JO. DELLY: structural variant discovery by integrated paired-end and split-read analysis. Bioinformatics. 2012;28: i333–i339. 10.1093/bioinformatics/bts378 22962449 PMC3436805

[30] Bankevich A, Nurk S, Antipov D, Gurevich AA, Dvorkin M, Kulikov AS, et al SPAdes: A New Genome Assembly Algorithm and Its Applications to Single-Cell Sequencing. J Comput Biol. 2012;19: 455–477. 10.1089/cmb.2012.0021 22506599 PMC3342519

[31] Husemann P, Stoye J. r2cat: synteny plots and comparative assembly. Bioinformatics. 2010;26: 570–571. 10.1093/bioinformatics/btp690 20015948 PMC2820676

[32] Darling AE, Mau B, Perna NT. progressiveMauve: Multiple Genome Alignment with Gene Gain, Loss and Rearrangement. PLoS ONE. 2010;5: e11147 10.1371/journal.pone.0011147 20593022 PMC2892488

[33] Tamura K, Stecher G, Peterson D, Filipski A, Kumar S. MEGA6: Molecular Evolutionary Genetics Analysis Version 6.0. Mol Biol Evol. 2013;30: 2725–2729. 10.1093/molbev/mst197 24132122 PMC3840312

[34] Drummond AJ, Suchard MA, Xie D, Rambaut A. Bayesian Phylogenetics with BEAUti and the BEAST 1.7. Mol Biol Evol. 2012;29: 1969–1973. 10.1093/molbev/mss075 22367748 PMC3408070

[35] Reyes JF, Chan CHS, Tanaka MM. Impact of homoplasy on variable numbers of tandem repeats and spoligotypes in Mycobacterium tuberculosis. Infect Genet Evol. 2012;12: 811–818. 10.1016/j.meegid.2011.05.018 21683165

[36] Nakanishi N, Wada T, Arikawa K, Millet J, Rastogi N, Iwamoto T. Evolutionary robust SNPs reveal the misclassification of Mycobacterium tuberculosis Beijing family strains into sublineages. Infect Genet Evol. 2013;16: 174–177. 10.1016/j.meegid.2013.02.007 23438651

[37] Ford CB, Shah RR, Maeda MK, Gagneux S, Murray MB, Cohen T, et al Mycobacterium tuberculosis mutation rate estimates from different lineages predict substantial differences in the emergence of drug-resistant tuberculosis. Nat Genet. 2013;45: 784–790. 10.1038/ng.2656 23749189 PMC3777616

[38] Sandegren L, Groenheit R, Koivula T, Ghebremichael S, Advani A, Castro E, et al Genomic Stability over 9 Years of an Isoniazid Resistant Mycobacterium tuberculosis Outbreak Strain in Sweden. PLoS ONE. 2011;6: e16647 10.1371/journal.pone.0016647 21304944 PMC3031603

[39] Saunders NJ, Trivedi UH, Thomson ML, Doig C, Laurenson IF, Blaxter ML. Deep resequencing of serial sputum isolates of Mycobacterium tuberculosis during therapeutic failure due to poor compliance reveals stepwise mutation of key resistance genes on an otherwise stable genetic background. J Infect. 2011;62: 212–217. 10.1016/j.jinf.2011.01.003 21237201

[40] Bertels F, Silander OK, Pachkov M, Rainey PB, van Nimwegen E. Automated Reconstruction of Whole-Genome Phylogenies from Short-Sequence Reads. Mol Biol Evol. 2014;31: 1077–1088. 10.1093/molbev/msu088 24600054 PMC3995342

[41] Iqbal Z, Turner I, McVean G. High-throughput microbial population genomics using the Cortex variation assembler. Bioinformatics. 2013;29: 275–276. 10.1093/bioinformatics/bts673 23172865 PMC3546798

[42] Peterlongo P, Schnel N, Pisanti N, Sagot M-F, Lacroix V. Identifying SNPs without a Reference Genome by Comparing Raw Reads In: Chavez E, Lonardi S, editors. String Processing and Information Retrieval. Springer Berlin Heidelberg; 2010 pp. 147–158. Available: http://link.springer.com/chapter/10.1007/978-3-642-16321-0_14

[43] Lin PL, Ford CB, Coleman MT, Myers AJ, Gawande R, Ioerger T, et al Sterilization of granulomas is common in active and latent tuberculosis despite within-host variability in bacterial killing. Nat Med. 2014;20: 75–79. 10.1038/nm.3412 24336248 PMC3947310

[44] Cohen T, van Helden PD, Wilson D, Colijn C, McLaughlin MM, Abubakar I, et al Mixed-Strain Mycobacterium tuberculosis Infections and the Implications for Tuberculosis Treatment and Control. Clin Microbiol Rev. 2012;25: 708–719. 10.1128/CMR.00021-12 23034327 PMC3485752

[45] Lew JM, Kapopoulou A, Jones LM, Cole ST. TubercuList– 10 years after. Tuberculosis. 2011;91: 1–7. 10.1016/j.tube.2010.09.008 20980199

