# Supplementary material for: Identifying Likely Transmission Pathways within a 10-Year Community Outbreak of Tuberculosis by High-Depth Whole Genome Sequencing
Source: PLoS One. 2016 Mar 3;11(3):e0150550. doi: 10.1371/journal.pone.0150550 (PMC4777479; doi:10.1371/journal.pone.0150550)
Supplement: S1 Fig — Locations of all SNPs and indels found in ≥75% of reads. Deletions, or the absence of an insertion, are indicated with a single dash (-). Genome locations of variants are given for the reference strain H37Rv (second row). The top row indicates the percentage of reads containing the variant. Colour coding of variants is based on the earliest isolate in which they first appeared; with all variants appearing in that isolate coded the same colour. This initial colour is then maintained when these variants appear in subsequent isolates, in order to visualise patterns of SNP accumulation. (PDF) [file pone.0150550.s001.pdf]

c30	c16	c37	c9	c12	c17	c14	c8	c27	c26	c25	c24	c29	c31	c32	c21	c15	c5	c28	c7	c4	c2	H37Rv	Position in H37Rv	
G	G	G	G	G	G	G	G	G	G	G	A	G	G	G	G	G	G	G	G	G	G	G	154900	99%
G	G	G	G	A	G	G	G	G	G	G	G	G	G	G	G	G	G	G	G	G	G	G	173422	100%
T	T	T	T	T	T	T	T	T	T	T	T	T	C	T	T	T	T	T	T	T	T	T	241392	100%
-	-	-	-	-	-	-	-	-	-	-	-	-	-	-	-	A	-	-	-	-	-	-	473306^7	100%
C	C	C	C	C	C	C	T	C	C	C	C	C	C	C	C	C	C	C	C	C	C	C	852625	87%
A	A	A	A	A	A	A	A	G	G	G	G	G	G	G	G	G	G	G	G	G	G	A	1008303	100%
G	G	G	G	G	G	G	A	G	G	G	G	G	G	G	G	G	G	G	G	G	G	G	1455790	87%
C	C	C	C	C	T	C	C	C	C	C	C	C	C	C	C	C	C	C	C	C	C	C	1669219	100%
G	C	C	C	C	C	C	C	C	C	C	C	C	C	C	C	C	C	C	C	C	C	C	1761523	99%
C	C	C	C	C	A	C	C	C	C	C	C	C	C	C	C	C	C	C	C	C	C	C	1778869	99%
G	G	G	G	G	G	G	G	T	G	G	G	G	G	G	G	G	G	G	G	G	G	G	1891805	93%
G	G	G	G	G	G	G	G	G	G	G	C	G	G	G	G	G	G	G	G	G	G	G	2053258	100%
G	G	G	G	G	C	G	G	G	G	G	G	G	G	G	G	G	G	G	G	G	G	G	2184243	100%
G	G	G	G	A	A	A	A	A	A	A	A	A	A	A	A	A	A	A	A	A	A	G	2320645	100%
T	T	T	T	C	C	C	C	C	C	C	C	C	C	C	C	C	C	C	C	C	C	C	2502667	100%
A	A	A	A	C	C	C	C	C	C	C	C	C	C	C	C	C	C	C	C	C	C	A	2566036	100%
C	C	C	C	C	T	T	C	C	C	C	C	C	C	C	C	C	C	C	C	C	C	C	2662735	100%
G	G	G	G	G	G	G	G	C	C	C	C	C	C	C	C	C	C	C	C	C	C	G	2716491	100%
C	C	C	C	A	C	C	C	C	C	C	C	C	C	C	C	C	C	C	C	C	C	C	2881037	100%
-	-	-	-	C	C	C	C	C	C	C	C	C	C	C	C	C	C	C	C	C	C	C	3138179	100%
C	C	C	C	T	T	T	T	T	T	T	T	T	T	T	T	T	T	T	T	T	T	C	3218218	99%
C	C	C	C	C	C	C	C	T	C	C	C	C	C	C	C	C	C	C	C	C	C	C	3343411	96%
C	C	C	C	C	C	C	C	A	A	A	C	C	C	C	C	C	C	C	C	C	C	C	3387492	100%
C	C	C	C	C	C	C	C	C	C	C	C	C	C	C	C	C	-	C	C	C	C	C	3494258	100%
C	G	G	G	G	G	G	G	G	G	G	G	G	G	G	G	G	G	G	G	G	G	G	3538087	100%
T	T	T	T	T	T	T	T	T	T	T	C	T	T	T	T	T	T	T	T	T	T	T	3690337	83%
G	G	G	G	G	G	G	G	G	G	G	G	T	G	G	G	G	G	G	G	G	G	G	3719370	99%
A	A	A	A	A	A	A	A	A	A	A	A	A	A	G	A	A	A	A	A	A	A	A	3773850	100%
G	G	G	A	G	G	G	G	G	G	G	G	G	G	G	G	G	G	G	G	G	G	G	3784904	79%
C	C	C	A	C	C	C	C	C	C	C	C	C	C	C	C	C	C	C	C	C	C	C	3792975	79%
A	G	G	G	G	G	G	G	G	G	G	G	G	G	G	G	G	G	G	G	G	G	G	3867072	100%
G	G	G	G	G	A	G	G	G	G	G	G	G	G	G	G	G	G	G	G	G	G	G	3952516	100%
T	T	T	T	T	T	T	T	T	T	T	T	C	T	T	T	T	T	T	T	T	T	T	4318153	100%
C	C	C	C	C	C	C	C	C	C	C	C	C	C	C	A	C	C	C	C	C	C	C	4323153	99%
