# Supplementary figures and images for: Identifying Likely Transmission Pathways within a 10-Year Community Outbreak of Tuberculosis by High-Depth Whole Genome Sequencing
Source: PLoS One. 2016 Mar 3;11(3):e0150550. doi: 10.1371/journal.pone.0150550 (PMC4777479; doi:10.1371/journal.pone.0150550)

a)

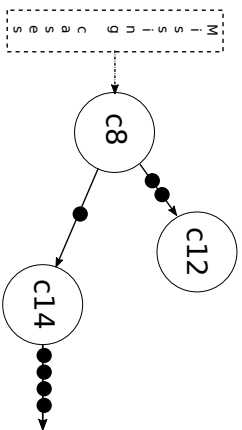

b)

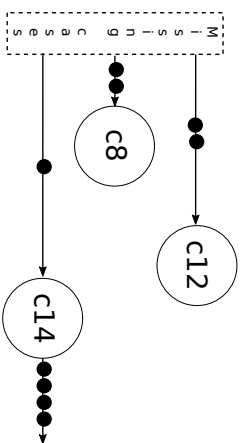

c)

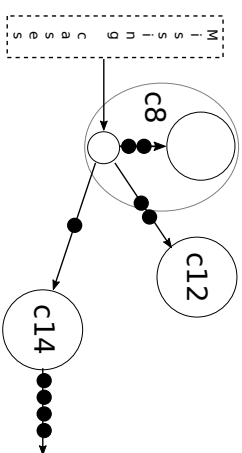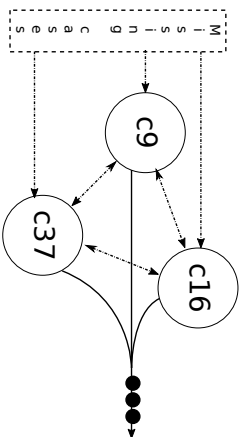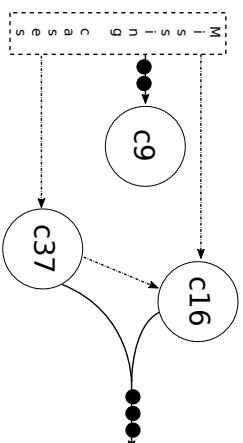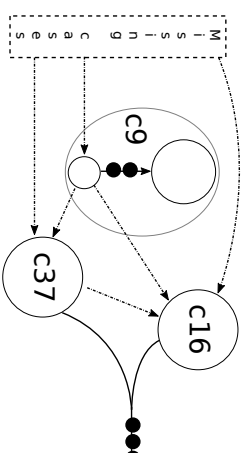

Supplement: S2 Fig — Differences in the transmission links for isolates c8 and c9 based on SNP profiles generated using (A) 90% SNP frequency threshold, corresponding to Fig 1, and (B) 75% SNP frequency threshold, corresponding to S1 Fig. The relative frequencies of SNPs detected suggested that several of the isolates in this cluster contained distinct subpopulations. Isolates c8 and c9 are shown to contain multiple variant subpopulations (C). One subpopulation forms part of a transmission pathway while the other subpopulation(s) do not. (PDF) [file pone.0150550.s002.pdf]
