# Supplementary material for: Identifying Likely Transmission Pathways within a 10-Year Community Outbreak of Tuberculosis by High-Depth Whole Genome Sequencing
Source: PLoS One. 2016 Mar 3;11(3):e0150550. doi: 10.1371/journal.pone.0150550 (PMC4777479; doi:10.1371/journal.pone.0150550)
Supplement: S1 Table — (PDF) [file pone.0150550.s003.pdf]

Genome	Position	ID	Reference	Alternative	Read depth	Allele Frequency
AL123456	36236	c28	C	T	262	0.62
AL123456	81122	c7	G	A	554	0.14
AL123456	86571	c14	C	T	321	0.11
AL123456	123878	c15	C	T	746	0.10
AL123456	480452	c37	C	G	374	0.11
AL123456	496344	c27	A	G	120	0.14
AL123456	556928	c25	C	A	308	0.13
AL123456	852625	c8	C	T	465	0.87
AL123456	853314	c8	C	T	483	0.11
AL123456	853672	c37	C	A	424	0.15
AL123456	998390	c16	G	T	303	0.14
AL123456	1131638	c8	C	T	465	0.11
AL123456	1273808	c15	G	A	695	0.22
AL123456	1455790	c8	G	A	407	0.86
AL123456	1464388	c16	A	G	344	0.13
AL123456	2329555	c2	C	T	840	0.20
AL123456	2377212	c16	G	T	325	0.11
AL123456	3029035	c9	G	A	249	0.11
AL123456	3165019	c28	G	A	359	0.13
AL123456	3277616	c7	C	T	514	0.13
AL123456	3690337	c24	T	C	319	0.82
AL123456	3968272	c7	G	A	605	0.11
AL123456	4104760	c16	A	C	479	0.46
AL123456	4338229	c8	A	G	440	0.35
AL123456	4317062	c29	T	C	663	1.00
AL123456	4317062	c28	T	C	507	0.40
AL123456	1761523	c9	C	G	260	0.13
AL123456	1761523	c30	C	G	732	0.99
AL123456	1669219	c14	C	T	356	0.33
AL123456	1669219	c17	C	T	502	1.00
AL123456	3952516	c14	G	A	381	0.10
AL123456	3952516	c17	G	A	530	1.00
