# Supplementary material for: Identifying Likely Transmission Pathways within a 10-Year Community Outbreak of Tuberculosis by High-Depth Whole Genome Sequencing
Source: PLoS One. 2016 Mar 3;11(3):e0150550. doi: 10.1371/journal.pone.0150550 (PMC4777479; doi:10.1371/journal.pone.0150550)
Supplement: S2 Table — (PDF) [file pone.0150550.s004.pdf]

Position in H37Rv	Ref	Alt	# Isolates	S/NS	% Reads	Rv	Gene/protein	Function	Other	Functional category (TubercuList)	Other notes (TubercuList)	Mutation - Himar1-based transposon mutagenesis of H37Rv (TubercuList)
154900	G	A	1	S	98.5	Rv0127	mak	maltokinase		Virulence, detoxification, adaptation	Catalyzes the transfer of a phosphate group from ATP to maltose to produce maltose-1-phosphate [catalytic activity: ATP + maltose = ADP + maltose-1-phosphate]	Essential for in vitro growth
173422	G	A	1	NS	99.8	Rv0147	calB	Coniferal aldehyde dehydrogenase (NAD+)		Intermediary metabolism and respiration	Function unknown; probably involved in cellular metabolism [catalytic activity: an aldehyde + NAD+ + H2O = an acid + NADH].	Non-essential for in vitro growth; partially or completely deleted in some clinical isolates
241392	T	C	1		99.5			Intergenic region	103 bp at 5' side: mmpl11; 122 bp at 3' side: exported protein			
473306	-	A	1	frameshift	98.0	Rv0393		Unknown	Conserved 13E12 repeat family protein	Insertion seqs and phages		Non-essential
852625	C	T	1	NS	86.8	Rv0757	phoP	Sensor-histidine kinase		Regulatory proteins	Possible PhoP - thought to be positive regulator for phosphate regulon, required for intracellular growth	Essential for in vitro growth, attenuated in human and murine macrophages
1008303	A	G	14	NS	99.9	Rv0905	echA6	enoyl-CoA hydratase EchA6		Lipid metabolism	Could possibly oxidize fatty acids using specific components [catalytic activity: (3S)-3-hydroxyacyl-CoA = trans-2(or 3)-enoyl-CoA + H(2)O].	Non-essential for in vitro growth
1455790	G	A	1	NS	86.7	Rv1299	prfA	Peptide chain release factor 1		Information pathways	Peptide chain release factor 1 directs the termination of translation in response to the peptide chain termination codons UAG and UAA	Essential for growth in vitro
1669219	C	T	1		99.5			Intergenic region	75 bp 5' side: inv1 - invasion associated protein; 64 bp 3' side: moxR1 transcriptional regulator			
1761523	C	G	1	NS	99.3	Rv1556		TetR-family regulatory protein		Regulatory proteins	Possibly involved in a transcriptional mechanism	Non-essential for in vitro growth
1778869	C	A	1	NS	99.4	Rv1571		Unknown	Conserved protein	Intermediary metabolism and respiration?	Conserved hypothetical, prediction based on GO and InterPro: intermediary metabolism and respiration	
1891805	G	T	1	NS	93.4	Rv1665	pks11	Chalcone synthase		Lipid metabolism	Possibly involved in the biosynthesis of secondary metabolites [catalytic activity: 3 malonyl-CoA + 4-coumaroyl-CoA = 4 CoA + naringenin chalcone + 3 CO2]	Non-essential for in vitro growth; mutant produces PDIM but lacks unknown polar lipids
2053258	G	C	1	NS	99.7	Rv1810		Hypothetical protein	DUF732: family consists of several uncharacterized M. tuberculosis and leprae proteins of unknown function	Conserved hypotheticals	Function unknown; at end of large PPE operon	Non-essential for in vitro growth
2184243	G	C	1	NS	100.0	Rv1933c	fadE18	Acetyl-coA dehydrogenase		Lipid metabolism	Function unknown, but supposed involvement in lipid degradation.	Non-essential for in vitro growth
2320645	G	A	18	S	99.6	Rv2062c	cobN	Cobalamin biosynthesis protein	Cobaltochelatase enzyme	Intermediary metabolism and respiration	Required for cobalt insertion	Non-essential
2502667	C	T	4	S	100.0	Rv2228c		Bifunctional RNase H/ acid phospatase		Information pathways	Unknown function; has RNaseH, alpha-ribazole phosphatase and acid phosphatase activities	Essential for in vitro growth (but not in CDC1551)
2566036	A	C	18	NS	99.6	Rv2294		Unknown	Probable aminotransferase	Intermediary metabolism and respiration	Unknown function; probable aminotransferase	Non-essential for in vitro growth
2662735	C	T	2	NS	99.5	Rv2380c	mbtE	Dimodular non-ribosomal peptide synthetase	Contains repeat domains	Lipid metabolism	Involved in the biogenesis of the hydroxyphenyloxazoline-containing siderophore mycobactins. Probably activates the two lysine residues that are incorporated into mycobactin (lysine ligation).	Essential for in vitro growth (but not in CDC1551)
2716491	G	C	14	S	100.0	Rv2418c		Unknown		Conserved hypotheticals	Conserved hypothetical, prediction based on GO and InterPro: intermediary metabolism and respiration	Essential for in vitro growth
2881037	C	A	1	S	100.0	Rv2560		Very end of proline and glycine rich transmembrane protein		Cell wall and cell processes	Unknown function, probably proline and glycine rich transmembrane protein	Non-essential for growth in vitro
3138179	C	-	4	frameshift	96.4	Rv2832c	ugpC	ABC transporter family	frameshift near C-terminus	Cell wall and cell processes	Active transport (import) of Sn-glycerol-3-phosphate across membrane	Essential for in vitro growth
3218218	C	T	18	NS	99.2	Rv2910c		Unknown		Conserved hypotheticals	Conserved hypothetical, prediction based on GO and InterPro: information pathways	Non-essential for growth in vitro
3343411	C	T	1	NS	96.4	Rv2986c	hupB	DNA-binding protein	Histone-like protein	Information pathways	Belongs to histone-like family of prokaryotic DNA-binding proteins capable of wrapping DNA to stablize it, preventing denaturation under extreme environmental conditions - transcription induced by starvation	Essential for in vitro growth
3387492	C	A	3	NS	100.0	Rv3028c	fixB	Electron transport flavoprotein alpha subunit	Energy production and conversion	Intermediary metabolism and respiration	The electron transfer flavoprotein serves as a specific electron acceptor for other dehydrogenases. It transfers the electrons to the main respiratory chain via ETF-ubiquinone oxidoreductase (ETF dehydrogenase).	Essential for in vitro growth
3538087	G	C	1	NS	99.6	Rv3169		Hypothetical protein	No putative conserved domains	Conserved hypotheticals	Unknown function and product	Non-essential for in vitro growth
3690337	T	C	1	NS	82.7	Rv3303c	lpdA	NAD(P)H quinone reductase		Intermediary metabolism and respiration	Involved in energy metabolism. Can catalyze the reduction of electron acceptors such as 2,6-dimethyl-1,4-benzoquinone (DMBQ) and 5-hydroxy-1,4-napthaquinone (5-HNQ) [catalytic activity: NAD(P)H + a quinone + H+ -> a quinol + NAD(P)+].	Conflicting studies: Essential and non-essential
3719370	G	T	1	NS	99.2	Rv3332	nagA	N-acetylglucosamine-6-phosphate deacetylase	Catalyses first committed step in peptidoglycan and teichoic acid synthesis	Cell wall and cell processes	Involved in N-acetyl glucosamine utilization pathway [catalytic activity: N-acetyl-D-glucosamine 6-phosphate + H(2)O = D-glucosamine 6-phosphate + acetate].	Conflicting studies: Essential and non-essential
3773850	A	G	1	S	99.6	Rv3362c		Hypothetical protein	Probable ATP/GTP binding protein	Conserved hypotheticals	Unknown function; probable ATP/GTP-binding protein	Non-essential
3784904	G	A	1		79.1			Intergenic region	164 bp 5' side: dnaE2 (DNA polymerase); 28 bp 3' side: putative diacyglycerol O-acetyltransferase			
3792975	C	A	1	NS	79.5	Rv3378c		Isotuberculosinol synthase	Product = diterpine synthase	Conserved hypotheticals	Produces isotuberculosinol (nosyberkol) from halimadienyl diphosphate. Production of tuberculosinol has also been shown by some.	Essential for in vitro growth; mutant shows reduced survival in macrophages
3867072	G	A	1	S	99.7	Rv3447c	eccC4	Unknown	ESX-4 Type VII secretion system protein	Conserved	Probable membrane protein. Could hydrolyse ATP/GTP.	Non-essential for in vitro growth
3952516	G	A	1		99.8			Intergenic region	19 bp 5' side: hypothetical protein; 19 bp 3' side: paaF (Rv3516)	(Rv3516 = immediately downstream - lipid metabolism)	(Rv3516 - could oxidize fatty acides using specific components)	(non-essential)
4318153	T	C	1		99.5			Intergenic region	988 bp 5' side: MTS2975 (putative small regulatory RNA); 619 bp 3' side of Rv3844 (putative transposase)			
4323153	C	A	1	S	98.9	Rv3848		Hypothetical protein	Transmembrane protein	Cell wall and cell processes	Unknown function; probable conserved transmembrane protein	No information
